# The Affymetrix DMET Plus Platform Reveals Unique Distribution of ADME-Related Variants in Ethnic Arabs

**DOI:** 10.1155/2015/542543

**Published:** 2015-02-22

**Authors:** Salma M. Wakil, Cao Nguyen, Nzioka P. Muiya, Editha Andres, Agnieszka Lykowska-Tarnowska, Batoul Baz, Asma I. Tahir, Brian F. Meyer, Grant Morahan, Nduna Dzimiri

**Affiliations:** ^1^King Faisal Specialist Hospital and Research Centre, Riyadh 11211, Saudi Arabia; ^2^The University of Western Australia, Perth, WA, Australia

## Abstract

*Background*. The Affymetrix Drug Metabolizing Enzymes and Transporters (DMET) Plus Premier Pack has been designed to genotype 1936 gene variants thought to be essential for screening patients in personalized drug therapy. These variants include the cytochrome P450s (CYP450s), the key metabolizing enzymes, many other enzymes involved in phase I and phase II pharmacokinetic reactions, and signaling mediators associated with variability in clinical response to numerous drugs not only among individuals, but also between ethnic populations.* Materials and Methods*. We genotyped 600 Saudi individuals for 1936 variants on the DMET platform to evaluate their clinical potential in personalized medicine in ethnic Arabs.* Results*. Approximately 49% each of the 437 CYP450 variants, 56% of the 581 transporters, 56% of 419 transferases, 48% of the 104 dehydrogenases, and 58% of the remaining 390 variants were detected. Several variants, such as rs3740071, rs6193, rs258751, rs6199, rs11568421, and rs8187797, exhibited significantly either higher or lower minor allele frequencies (MAFs) than those in other ethnic groups.* Discussion*. The present study revealed some unique distribution trends for several variants in Arabs, which displayed partly inverse allelic prevalence compared to other ethnic populations. The results point therefore to the need to verify and ascertain the prevalence of a variant as a prerequisite for engaging it in clinical routine screening in personalized medicine in any given population.

## 1. Introduction

The response of an individual to drug therapy is determined by many variables, including food intake, age, and most importantly genetic factors. It is now well acknowledged that alterations in genes encoding drug metabolizing enzymes, ion transporters, and receptors, among others, have a great influence on the pharmacokinetics and pharmacodynamics of therapeutic agents, triggering variations in patient response to drug therapy. By far the largest of such gene families is that encoding the cytochrome P450 (CYP450) superfamily of enzymes, a diverse group of enzymes that catalyze the oxidation of organic substrates, including metabolic intermediates, such as lipids and steroidal hormones, and xenobiotics, such as drugs and other toxic substances [1–4]. The CYP450s are the major enzymes involved in about 75% of drug metabolism and bioactivation. Thereby, the CYP450 3A (CYP3A) isoforms are thought to be responsible for metabolizing approximately 55%, CYP2D6 about 30%, and CYP2C about 15–20% of the drugs [5].

Apart from the CYP450 metabolizers, other protein families involved in the absorption, distribution, metabolism, and elimination (ADME) of therapeutic agents include transporters, transferases, dehydrogenases, monooxygenases, reductases, and receptors. Transporter gene families include the adenosine triphosphate-binding cassette (ABC) and solute transporters (SLC) [6–8], while transferases comprise the glucuronosyltransferases (UGTs), glutathione S transferases (GSTs), sulfotransferases (SULTs), and catechol-O-methyl transferase (COMT) that may also determine the fate of a drug [9–11]. Other ADME-related gene families include those encoding dehydrogenases (DHO), monooxygenases, and reductases [12, 13]. Besides, changes in some receptor families, such as the nuclear receptor subfamilies, *β*-adrenergic receptors, and peroxisome proliferator-activated receptor subtypes, just to name a couple, also contribute to variations in the ways by which patients respond to drug therapy [14–17].

It is now well established that patient response to drug therapy will depend on the genetic structure of the enzyme or protein involved in its metabolism, and the allelic frequencies and phenotypic consequences may vary considerably between ethnic groups [18–22]. Hence, great effort has been directed at exploiting our knowledge of changes in these genes for clinical purposes in personalized medicine. One of these endeavors is the development of the Affymetrix DMET Plus array which facilitates the highly multiplexed genotyping of known polymorphisms of a panel of markers from 225 ADME-related genes on a single array. These variants have been documented to be of importance in phase I and phase II drug metabolism and disease manifestation. While this platform has found its place in personalized medicine, it has also become increasingly apparent that the influence of these genetic changes varies not only among individuals, but also between ethnical populations. This renders it necessary to acquire adequate prior knowledge of the likely impact of such entities, if they were to be considered for routine clinical purposes in any given society. Currently only isolated data is available on the prevalence of the gene variants in ethnic Arabs. Hence, this study was designed to characterize the prevalence of these variants with the focus on establishing their relevance in personalized management of various indications with different types of drugs in ethnic Arabs, employing the Saudi Arab population as a homogeneous study model.

## 2. Methods

### 2.1. Patient Sample Collection

The study candidates comprised 600 individuals randomly drawn from our coronary artery disease (CAD) registry who were subjected to genotyping by the DMET Plus chip. This registry contains affected as well as nonaffected individuals. All individuals signed an informed consent, and the study protocol was approved by the Institutional Review Board (IRB) at the King Faisal Specialist Hospital and Research Centre. Genomic DNA was extracted by a standard phenol extraction procedure.

### 2.2. Genotyping by Affymetrix DMET Plus Array

The genotyping was accomplished by the DMET (Drug Metabolizing Enzymes and Transporters) Plus Premier Pack, a microarray assay developed by Affymetrix (Affymetrix, Santa Clara, CA, USA) designed specifically to test drug metabolism associations. The DMET array contains 1936 (1931 SNPs and 5 CNVs) drug metabolism markers in 225 genes including 47 phase I enzymes, 80 phase II enzymes, 52 transporters, and 46 other genes. These genetic variants were multiplex genotyped using the molecular inversion probe (MIP) technology [23, 24]. Briefly, some markers from regions containing pseudogenes and close homologs were first preamplified using a multiplex polymerase chain reaction (mPCR) (Qiagen, Valencia, CA, USA). Genomic sequences that contain the polymorphic markers of interest were then preferentially amplified through the use of highly selective MIPs. A first quality control (QC) gel was run to determine the quality of amplified MIPs, which should be a single band represented on a gel in the range of 100–150 base pairs. Smaller DNA fragments were generated by adding fragmentation reagents to improve sample hybridization with the DMET Plus array, and DNA fragment size was checked on the second QC gel, in which the fragment length should be less than 120 base pairs with a smear centered at approximately 50 base pairs. The resulting target DNA was then labelled and hybridized to the DMET Plus array to obtain genotypes using a single color detection format. The profiles for the genotyping call rates and concordance comparisons were generated by the DMET Console software which is based on the BRLMM (Bayesian Robust Linear Model with Mahalanobis distance classifier) algorithm. Fixed genotype boundaries were used as the algorithms for all genotyping configurations. Genotypes were reported as homozygous wild type, heterozygous, homozygous variant or “no call.” CNV markers and SNPS with call rate less than 100% were excluded from the subsequent analysis.

## 3. Results

**Figure 1:**
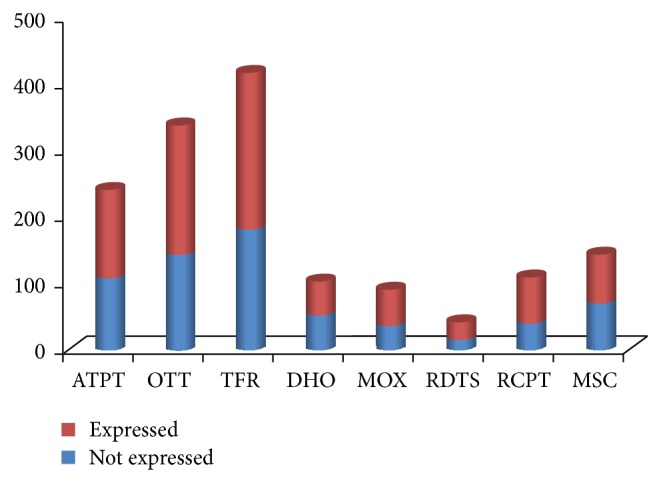
Comparison of the detected versus non-detected variants by the functional groups of the gene families. ATPT, ATP transporters; OTT, other transporters; TFR, transferases; DHO, dehydrogenases; MOX, monooxygenases; RDTS, reductases; RCPT, receptors; MSC, miscellaneous gene families.

The present study genotyped 600 Saudi individuals for the 1936 gene variants involved in drug absorption, distribution, metabolism, and elimination (ADME), with documented functional significance in phase I and II drug metabolism, as well as pharmacodynamics of several therapeutic agents. The gene variants, which include transporters, ion transferases, and receptors, displayed various distribution profiles.  Figure 1 summarizes the profiles of the different gene variants by functional groups. As indicated in this figure, overall some 877 (45%) displayed no allelic change at all in our study population.

**Figure 2:**
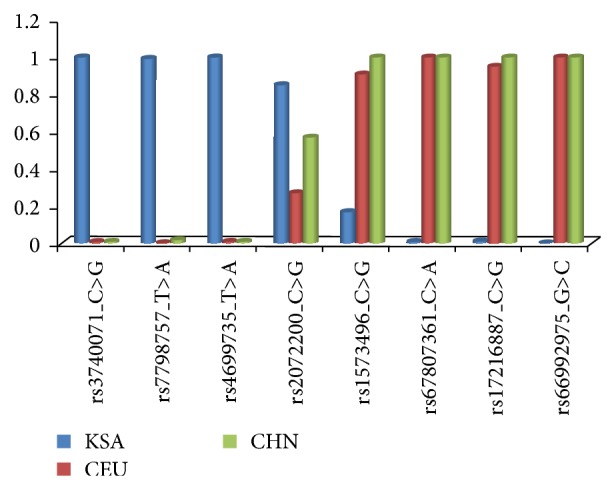
Examples of SNPs displaying an inverse relationship of the allele frequencies in the minor alleles in Caucasians (CEU) and Chinese (CHN) and ethnic Saudi Arab populations (KSA).

By far the largest number of SNPs on the DMET platform belongs to the drug metabolizing superfamilies, whereby the CYPs constitute the majority. This superfamily comprised about 437 SNPs, of which the CYP2C (53), CYP3A (53), CYP1A (30), and CYP2D (30) constitute the major subfamilies (Table 1). When classified by subfamilies, the data revealed that about 51% of the CYP2C, 32% of the CYP3A, 33% of CYP1A, and 53% of CYP2D variants (Table 1) displayed detectable minor alleles. Furthermore, while, in the majority of the cases, the minor allele frequencies (MAFs) fell in the range of 0.001–0.5, noticeable variations were observed among the family members. To begin with, as depicted in Figure 2, several SNPs exhibited an inverse distributional profile compared to available databases on other populations, such as the Caucasians or Chinese (see DMET Supplementary Data in Supplementary Material available online at http://dx.doi.org/10.1155/2015/542543). Thus, our population displays a whole profile range from variants, such as rs2072200_C>G or rs1573496_C>G in which the minor allele in other ethnic populations turned to be the major allele or vice versa in our population, to those lacking any genetic change such as rs3740071_C>G, rs8187797_C>G, or rs11568421_G>A in our population as opposed to others (Figure 2). Besides, several of the variants displayed either marginally or significantly greater MAFs than those in other ethnical populations, with sizeable number exhibiting MAFs >0.49 (DMET Supplementary Data). Notably, while several variants found in other populations in some smaller CYP gene subfamilies, such as CYP11A, CYP21A, and CYP46A, were not at all detected, those in the CYP2F, CYP4B, CYP7B, CYP8B, and CYP26A subsets displayed discernible minor alleles (Table 1; DMET Supplementary Data).

In addition to the metabolizing CYPs, the other large groups of ADME-related variants included the transporters such as the SLCs comprising 322 variants, of which 58% were detectable and the ABCs comprising 242 variants of which 56% were detected in our population. The partial lack of change was also evident among other superfamilies of transporters, transferases, dehydrogenases, monooxygenases, reductases, receptors, and other signalling entities (Figure 1; Table 2). In summary, we were also unable to detect approximately 41% of the variants in other major ADME gene families, including the ABCs, SLCs, SULTs, GSTs, and PPARs.

Apart from the transporters, the platform also carries several families of transferases, including the uridine diphosphate glucuronosyltransferases (UGTs), SULTs, glutathione S-transferase alpha (GSTA), glutathione S-transferase Mu (GSTM), glutathione S-transferase omega (GSTO), glutathione S-transferase pi (GSTP), histamine N-methyltransferase, methionine adenosyl transferase (MAT), N-acetyl glucosamine transferase, nicotinamide N-methyltransferase, thiopurine S-methyltransferase (NNMT), COMT, phenylethanolamine N-methyltransferase (PNMT), and quinolinate phosphoribosyltransferase (QPRT). The largest transferase gene superfamily studied was that of the microsomal UGTs, composed of at least 16 genes responsible for the elimination of a myriad of xenobiotics and endogenous compounds. Of the 116 UGT variants, only 50% showed detectable changes (Table 2). Furthermore, we were also able to detect 67% of the 138 SULTs, 52% of the 88 GSTs, and majority of the others.

Aldehyde dehydrogenases (ALDH; 50) and alcohol dehydrogenases (ADHs; 40) similarly constituted the greater part of the 111 DHO family. Other DHOs included six glucose 6-phosphate dehydrogenase (G6PD), seven nicotinamide phosphate (quinone) dehydrogenases (NQOs), and eight xanthine dehydrogenases (XDHs). Altogether 54 (52%) of the DHOs did not show any changes. Furthermore, monooxygenases, such as FMOs or MAOs, constitute a group of 92 variants, of which about 54% were detectable (Table 2). Besides, about 64% of the studied receptors and other signaling gene variants were detectable, including 21 for ralA binding protein 1 (RALBP1), 12 for nuclear receptor subfamily 1, group I, members 2 and 3 (NR1l2 and NR1l3), 53 for PPARs, and 12 for nuclear receptor subfamily 3, group C, member 1 (NR3C), retinoid X receptor, alpha (RXRA) nuclear receptor subfamily 2, group B member, to name a few. Also, approximately 61% of these SNPs showed detectable minor alleles in the present study (Table 2).

Put together, we were able to detect minor alleles for 49% of the CYPs, 57% of the transporters, and 56% of the transferases on the DMET platform in our study population. Of the rest of the gene variants, we were unable to detect minor alleles for 43%. Among these SNPs, 72 showed MAF values of >0.45, whereby 18 displayed values of >0.49.

### 3.1. Statistical Analysis

Comparison of genotypes and alleles between different groups for continuous dependent variables was accomplished by analysis of variance (ANOVA) or Student's* t*-test as appropriate. Categorical variables were analyzed by Chi-Square test, and logistic regression analysis was used to compute odds ratios and their 95% confidence intervals. All other statistical analyses were performed using the SPSS software version 14 (SPSS Inc., Chicago, USA). Associations with a two-tailed *P* value < 0.05 were considered statistically significant.

## 4. Discussion

The present study established the prevalence of the 1936 DMET Plus platform variants in several gene families involved in the pharmacokinetics and pharmacodynamics of several important therapeutic agents for different ailments. We detected approximately 55% of the SNPs on this platform, pointing to the fact that only a portion of them is likely to be economically worthwhile pursuing in personalized medicine in ethnic Arabs. Currently, there is great lack of data on the distribution of these ADME variants in this population. In fact, to the best of our knowledge, this is the first and largest study reporting their prevalence in an Arab population. This data should therefore serve as a basis for evaluating the usefulness of routinely assaying these SNPs for clinical purposes in this ethnic group.

Perhaps the most widely studied family of metabolizing enzymes is the CYP superfamily. These enzymes display a wide range of phenotypes from poor, rapid, to ultrarapid metabolizers for several important agents, due to the variations in the combinations of their encoding alleles. An example is that of the CYP2C19 with more than 19 variants encoding the nonfunctional* CYP*2C19^*^2 and inactive enzyme* CYP*2C19^*^3, on one hand, and an ultrarapid metabolizing* CYP*2C19^*^17 and extensive metabolizer phenotype* CYP2C19* ^*^ *1 *on the other hand [4, 25, 26]. Accordingly, poor metabolizers of drugs that are processed through the CYP2C19 pathways frequently experience dramatic changes in drug responses and side effects when they receive standard doses. Thus, for example, the CYP2C19^*^2 loss-of-function allele has been associated with a decreased activation of clopidogrel [27, 28], attenuation of its antiplatelet effect [29–35], and contributing to 3- to 6-fold incidence of stent thrombosis in patients treated with percutaneous coronary intervention (PCI) [29–35], while the presence of any gain-of-function* CYP2C19* ^*^17 has also been linked to increased risk of bleeding [36]. Given these potential clinical consequences of harbouring the* CYP2C19* gene variants that may affect therapeutic modalities, it is not surprising that a surge of attempts has grown exponentially in recent years to employ this knowledge clinically in personalized medicine. Besides, some researchers have suggested a link between CYP2C19 polymorphisms and diseases, such as digestive tract cancer [37] and essential hypertension [38]. However, their phenotypic expression has been studied primarily in Caucasians [20] and some other ethnic populations, but only poorly so in Arabs. In fact, very limited information is currently available on the prevalence of these variants in the Saudi population, with only a couple of studies appearing recently on two variants, the CYP2C19^*^2 and CYP2C19^*^3, albeit involving very small study populations [39, 40]. Hence, the establishment of their prevalence in the present study can be viewed as an important step in identifying the clinically relevant SNPs in this population. Specifically, our results indicate that it is worthwhile screening for the different CYP2C19 variants, for example, for such purposes in our population.

Like the CYP450s, several gene variants encoding other ADME-related proteins also exhibited diversity in their distribution, ranging from those that showed no changes, such as the rs6193_A>G and rs258751_G>A, to those that exhibited inverse profiles, such as rs3740071_C>G and rs17216887_C>G in the ABCs. To date, mutations in the ABCP have been associated with cancer chemotherapy drug resistance [41, 42], atherosclerosis, inflammation [43], and several other diseases [43–45], while disorders linked to ATP7A include Menkes disease and occipital horn syndrome [46, 47]. Hence, in our population, these variants may be relevant not only with respect to drug response, but also disease manifestation, and further studies are necessary to elucidate the extent of their impact on disease in this ethnic population.

Although much remains to be learnt about UGTs, a number of polymorphisms are thought to be of toxicological significance [48] or have been associated with diseases, such as Crigler–Najjar's and Gilbert's syndrome [49]. Thus, several of its SNPs, including the rs7586110 (UGT1A7^*^12_c.-57T>G) (MAF = 0.417) and rs8175347 (UGT1A1^*^28_c.TATA-box) (MAF = 0.271), have been previously linked to different types of diseases, including cancer, cardiovascular diseases, and irinotecan toxicity in patients with Gilbert's syndrome, to name a few [50–53]. Perhaps one of the best-studied transferase gene families is the* GST*, which also constitutes one of the largest groups of variants on the platform. The prevalence of the wide majority of the* GST*s was similar to that described in other ethnic groups, suggesting that their impact on disease is likely to be global. Furthermore, the study also revealed significant changes in the studied SULTs, which constitute the third largest family of transferases on the platform. Since SULTs can activate procarcinogens to reactive electrophiles [11], enzymes such as the steroid sulfatase and estrogen SULTs have been implicated in human carcinomas [54].

In addition to transporters and transferases, other ADME families including DHOs, monooxygenases, reductases, various receptors, and signal transducers on the platform also equally displayed diversity in the variant profiles. Put together, the data indicates that approximately 45% variants could not be detected in our study population. Furthermore, the majority of those that were detectable presented with MAF >0.01, with a sizeable portion being at variance with the data in the literature.

Since almost 50% of the loci were unchanged in the present population, it was of interest for us to compare the discovered profiles with those of other ethnic groups, as a test for the robustness of the DMET platform as a potential global clinical tool. As might be expected, our analysis revealed some similarities with other ethnic populations in the alterations of a number of variants. Thus, for example, the results point to relatively similar frequencies for the CYP2C19^*^2 (rs4244285) to those in several other ethnic groups, including the Romanian (0.12), Lebanese (0.13) [40], Turkish (0.12) [55–57], Jewish (0.15%) [39], Russian (0.11) [58], and Italian (0.12) [59], but slightly lower than those in the Chinese (0.25) [60, 61], North Indian (0.26) [62], and Thai (0.29) [22] populations. We also found low MAFs for CYP2C19^*^2 (0.093) and* CYP*2C19^*^3 (0.001) which were comparatively lower than in Africans, while that of the* CYP2C19* ^*^17 (0.256) matched those of the European populations but was higher than those in African and other Asian populations (see also Supplementary Data). More importantly, in depth analysis pointed to several SNPs, featuring conversions in which a minor allele in the European/Caucasian populations not only turned to be the major one but also exhibited no change in the Saudi population. On the other end of the spectrum were also several major alleles in European or Asian populations that could not be detected in our population. Thus, put together, the study demonstrates that although the distribution of most of these variants was within similar ranges with those in other populations, some distinct interethnical differences in the prevalence of many others were also evident between ethnic Arabs and other ethnic populations.

The important question arises as to the clinical relevance of these findings with respect to targeting the variants for personalized medicine. First, our observations stress the fact that not all ADME variants constitute therapeutically meaningful targets in the ethnic Arab population, as reflected by their absence in our study population. The wide interethnical variations in the prevalence of several of the variants supports the notion that the depth of involvement of these variants will also vary among different ethnic groups. Thus, for example, several genotypes that are otherwise lowly distributed in other ethnic population might be of great significance and vice versa, in this regard. In particular, the observations of inverse genotype relationships in which the alleles displayed literally the opposite level of expression, such as rs3740071 or rs4699735, might also imply, for example, that these variants will exert opposing effects on drug response in different ethnic groups. This, in turn, renders it practically impossible to generalize the mode by which such variants may influence therapeutic modalities globally and therefore necessitates acquisition of adequate knowledge of their prevalence in any given community prior to engaging them in targeted genotyping for clinical purposes in personalized medicine. Besides, our current findings further open the door to also critically evaluate the role of the studied gene variants in disease. Hence, their actual clinical impact on disease management needs to be revisited more closely.

In summary, the present study utilized the availability of the DMET Plus platform to estimate the prevalence of ADME-related variants of potential therapeutic relevance, using the Saudi population, as a basis for informed targeting of these variants in personalized medicine in ethnic Arabs. We were able to detect approximately half of the variants on this platform, not only reaffirming the prevalence of some important variants in our population, but also furnishing some support for the usefulness of the procedure in routinely detecting the presence of these genotypes for clinical purposes. More importantly, we observed some significant differences in the expression of several variants in comparison to other ethnic populations, laying the foundation for adopting evidence-based approaches to personalized medicine in ethnic Arabs.

## Supplementary Material

The supplementary material is a compilation of the genotyping data for the 1936 ADME-related gene variants in the Saudi Arab population and available literature of the their distribution in the Caucasian and Chinese populations (for comparison).

## Figures and Tables

**Table 1:** Distribution of some of the common CYP450 variants.

Group	Total	Undetected	<0.01	>0.01
All	437	222 (0.51)	55 (0.125)	160 (0.366)
CYP1A	30	20 (0.666)	1 (0.033)	9 (0.3)
CYP1B	20	16 (0.80)	1 (0.5)	3 (0.15)
CYP2A	34	20 (0.58)	3 (0.09)	11 (0.32)
CYP2B	24	10 (0.42)	2 (0.08)	12 (0.5)
CYP2C	53	26 (0.49)	14 (0.26)	13 (0.25)
CYP2D	30	14 (0.47)	4 (0.13)	12 (0.4)
CYP2E	10	2 (0.20)	3 (0.3)	5 (0.5)
CYP2F	5	0 (0.0)	2 (0.4)	3 (0.6)
CYP2J	9	8 (0.89)	0 (0.0)	1 (0.11)
CYP2S	4	1 (0.25)	1 (0.25)	2 (0.5)
CYP3A	53	36 (0.68)	3 (0.06)	14 (0.26)
CYP4A	4	1 (0.25)	1 (0.25)	2 (0.5)
CYP4B	7	0 (0.0)	2 (0.28)	5 (0.72)
CYP4F	35	12 (0.34)	2 (0.06)	21 (0.6)
CYP4Z	4	1 (0.25)	0 (0.0)	3 (0.75)
CYP7A	7	1 (0.142)	1 (0.142)	5 (0.714)
CYP7B	4	0 (0.0)	1 (0.25)	3 (0.75)
CYP8B	7	0 (0.0)	4 (0.571)	3 (0.428)
CYP11A	7	7 (1.00)	0 (0.0)	0 (0.0)
CYP11B	24	15 (0.63)	0 (0.0)	9 (0.36)
CYP17A	6	3 (0.50)	0 (0.0)	3 (0.50)
CYP19A	11	6 (0.54)	0 (0.0)	5 (0.46)
CYP20A	5	2 (0.40)	0 (0.0)	3 (0.60)
CYP21A	3	3 (1.00)	0 (0.0)	0 (0.0)
CYP24A	8	4 (0.50)	0 (0.0)	4 (0.50)
CYP26A	5	0 (0.0)	2 (0.4)	3 (0.6)
CYP26C	1	0 (0.0)	1 (1.0)	0 (0.0)
CYP27A	6	4 (0.67)	2 (0.33)	0 (0.0)
CYP27B	9	5 (0.56)	2 (0.22)	2 (0.22)
CYP39A	4	1 (0.25)	1 (1.25)	2 (0.50)
CYP46A	1	1 (1.00)	0 (0.0)	0 (0.0)
CYP51A	7	3 (0.43)	2 (0.285)	2 (0.285)

The table displays the relative minor allele frequency distribution of the CYP450 variants determined by DMET Plus chip in 600 individuals.

**Table 2:** Distribution levels for non-CYP450 variants on the DMET Plus platform.

Group	Type	All	MAF distribution		
			Undetected	<0.01	>0.01
Transporters	ABC transporters	242	108 (0.44)	33 (0.14)	101 (0.42)
	Solute transporters	322	137 (0.42)	47 (0.15)	138 (0.43)
	ATPase, Cu^2+^ transporters, alpha peptide	17	7 (0.41)	1 (0.06)	9 (0.53)

Transferases	Glucuronosyltransferases (UGTs)	116	58 (0.50)	11 (0.10)	47 (0.40)
	Sulfotransferases	138	45 (0.33)	18 (0.13)	75 (0.54)
	Glutathione S-transferase	88	42 (0.48)	4 (0.04)	42 (0.48)
	N-Acetyl glucosamine transferase	37	20 (0.54)	5 (0.13)	12 (0.32)
	Others (COMT, HNMT, MAT, NNMT, TPMT, QPRT, PNMT)	40	19 (0.48)	5 (0.12)	16 (0.40)

Dehydrogenases	ADHs; ALDHs, XDHs, G6PD	104	54 (0.52)	7 (0.07)	43 (0.41)

Monooxygenases	FMOs, MAOs, AOX	92	38 (0.41)	10 (0.11)	44 (0.48)

Reductases	VKORC1, HMGCRs, POR, CBR	43	16 (0.37)	7 (0.16)	20 (0.47)

Receptors	NR1, members 2 and 3	21	11 (0.52)	3 (0.14)	7 (0.33)
	PPAR, gamma, delta	53	13 (0.25)	2 (0.03)	38 (0.72)
	Nuclear receptor subfamily 3	12	5 (0.42)	5 (0.42)	2 (0.16)
	Others (RALBP1, SPN, APOA, RXRA, AHR, ARNT)	25	11 (0.44)	4 (0.16)	10 (0.40)

Miscellaneous	ALB, ARSA, CA5P AKAP, CDA, CES, PTGIS, CCDC, CROT, SPG, FAAH, EPHX, DCK, PON, TBXAS, TPSG, ORM, PGAP3, SERPINA, PPP1R9, PRSS53, RPL, TYMS	144	71 (0.49)	21 (0.15)	52 (0.36)

ABC, ATP-binding cassette transporters; ALDH, aldehyde dehydrogenase; ADH, alcohol dehydrogenase class 3; ALB, albumin; ARSA, arylsulfatase; AHR, aryl hydrocarbon receptor; AKAP, A-kinase anchor proteins; APOA, apolipoprotein receptor, subtype A; AOX, alternative oxidase; CA5P, carbonic anhydrase; CBR, carbonyl reductase; CCDC, coiled-coil domain-containing protein; CDA, cytidine deaminase; CES, carboxylesterase; COMT, catechol-O-methyl transferase; CROT, peroxisomal carnitine O-octanoyltransferase; DCK, deoxycytidine kinase; EPHX, microsomal epoxide hydrolase; FAAH, fatty acid amide hydrolase; FMO, flavin-containing monooxygenase; G6PD, glucose 6-phosphate dehydrogenase; HNMT, histamine N-methyltransferase; MAO, monoamine oxidase; MAT, methionine adenosyl transferases; NNMT, nicotinamide N-methyltransferase; NR1, nucleic acid receptor, group 1; ORM, orosomucoid; PGAP3, post-GPI attachment to proteins 3; PON, serum paraoxonase/arylesterase; POR, cytochrome P450 reductase; PPAR, peroxisome proliferator-activated receptors; PNMT, phenylethanolamine N-methyltransferase; PTGIS, prostaglandin-I synthase; QPRT, quinolinate phosphoribosyltransferase; PPP1R9, protein phosphatase 1, regulatory subunit 9B; PRSS53; serine protease, 53; RALBP1, ralA binding protein 1; RPL, ribosomal protein-binding receptor; RXRA, retinoic acid receptor; SERPINA, serpin peptidase inhibitor, clade A (alpha-1 antiproteinase, antitrypsin); SPG, spastic paraplegia; SPN, sialophorin receptor; TBXAS, thromboxane A synthase 1 (platelet); TYMS, thymidylate synthetase; TPMT, thiopurine S-methyltransferase; TPSG, tryptase; XDH, xanthine dehydrogenases.
